# EPHA5 mutations predict survival after immunotherapy in lung adenocarcinoma

**DOI:** 10.18632/aging.202169

**Published:** 2020-12-03

**Authors:** Zhiming Chen, Ji Chen, Dandan Ren, Jiao Zhang, Ying Yang, Henghui Zhang, Beibei Mao, Haitao Ma

**Affiliations:** 1 Department of Thoracic Surgery, The First Affiliated Hospital of Soochow University, Suzhou, Jiangsu Province, China; 2 Department of Thoracic Surgery, Huashan Hospital, Shanghai, China; 3 Genecast Precision Medicine Technology Institute, Beijing, China; 4 Institute of Infectious Diseases, Beijing Ditan Hospital, Capital Medical University, Beijing Key Laboratory of Emerging Infectious Diseases, Beijing, China

**Keywords:** immunotherapy, lung adenocarcinoma, EPHA5, TMB, PDL1

## Abstract

Eph receptors constitute the largest family of RTKs, and their associations with antitumor immunity and immunotherapy are largely unknown. By integrating genomic, transcriptomic and clinical data from cohorts in public databases, we identified EPHA5 as the most common mutated gene of Eph receptors in lung adenocarcinoma (LUAD). Moreover, compared with EPHA5 wild-type (WT) patients, EPHA5-mutant (Mut) patients exhibited significantly enhanced infiltration of CD8^+^ T cells and M1 macrophages, reduced recruitment of immunosuppressive regulatory T cells (Tregs) into the tumor site, as well as the increased level of chemokine, interferon-gamma, inhibitory immune checkpoint signatures, tumor mutation burden (TMB) and tumor neoantigen burden (TNB). Additionally, EPHA5 mutation cooccurred with homologous recombination (HR) or mismatch repair (MMR) gene mutations. These data were validated in the LUAD cell line H1299 and a Chinese LUAD cohort. Most importantly, clinical analysis of a Memorial Sloan Kettering Cancer Center (MSKCC) immunotherapy cohort indicated that LUAD patients with EPHA5 mutations who were treated with immunotherapy had markedly prolonged survival times.

Our results revealed the correlation of EPHA5 mutations with tumor immune microenvironment and predictive factors for immunotherapy, implying the potential of EPHA5 mutations as a prognostic marker for the prognosis of LUAD patients to immune checkpoint blockade therapy.

## INTRODUCTION

Lung cancer is the leading cause of cancer-related death worldwide [1] and is classified mainly as small cell lung cancer (SCLC) and non-SCLC (NSCLC). On the basis of histological parameters, NSCLC is classified as lung adenocarcinoma (LUAD), lung squamous cell carcinoma (LUSC) and large cell carcinoma.

Small molecule protein kinase inhibitors targeting tyrosine kinases, including epidermal growth factor receptor (EGFR) and anaplastic lymphoma kinase (ALK), have been widely used to improve the prognosis of advanced NSCLC patients with genetic alterations in these targeted genes [2, 3]. Patients expressing the wild-type (WT) form of these tyrosine kinases may derive therapeutic benefit from compounds that target immune checkpoints such as programmed death-1 (PD-1) and programmed death ligand-1 (PD-L1) [4]. For example, pembrolizumab, which targets PD-1, is approved by the Food and Drug Administration (FDA) for treating advanced NSCLC patients without mutations in EGFR or ALK and with high PD-L1 expression [5]. However, a large proportion of cancer patients do not respond to this therapy. Substantial evidence indicates that the response to cancer immunotherapy is closely related to PD-L1 expression [6], the tumor mutation burden (TMB) [7] and tumor-infiltrating lymphocytes (TILs) [8]. In addition, in NSCLC, mutations in specific genes may be correlated with the immunotherapy response, e.g., commutation of TP53 and KRAS improves the response of patients to anti-PD-1 therapy [9].

Eph receptors constitute the largest family of receptor tyrosine kinases (RTKs). In vertebrates, this family has 16 members, namely, EphA receptors 1-10 (EphA1-A10) and EphB receptors 1-6 (EphB1-B6) [10]. Upon binding of their Ephrin ligands, these receptors transduce external signals into cells through the Src, RAS/MAPK, and integrin pathways and control a variety of biological processes related to tumor progression [11–13]. Eph receptors are also expressed on cells of the immune system, and Eph-ephrin interactions have been reported to mediate immune cell activation, migration, adhesion, and proliferation [14–16]. However, publications regarding the relationship of Eph receptors withthe tumor immune microenvironment are limited. In a recent study, researchers found that knockout of EphA10 reduced the expression of PDL1 in breast cancer cells [17]. This finding prompted us to explore the relevance of Eph receptors to antitumor immunity in the tumor microenvironment (TME).

In this study, we performed an integrated analysis incorporating DNA sequencing and RNA sequencing (RNA-seq) data from the LUAD dataset in The Cancer Genome Atlas (TCGA) database and a Chinese cohort to determine the correlations of Eph receptor mutations with antitumor immunity in LUAD by a bioinformatics approach. We found that mutation of EPHA5, the most frequently mutated gene in the Eph receptor family, was associated with CD8^+^ T cell infiltration, intratumoral PD-L1 expression, increased TMB and mutations in DNA damage response (DDR) pathway genes. Significantly, we revealed that the survival of EPHA5-mutant (Mut) LUAD patients was more favorable than that of EPHA5-WT patients in an immunotherapy setting, providing new insight into the search for predictive biomarkers for the immunotherapy response in lung cancer.

## RESULTS

### EPHA5 mutations are associated with enhanced immunity in LUAD

**Figure 1:**
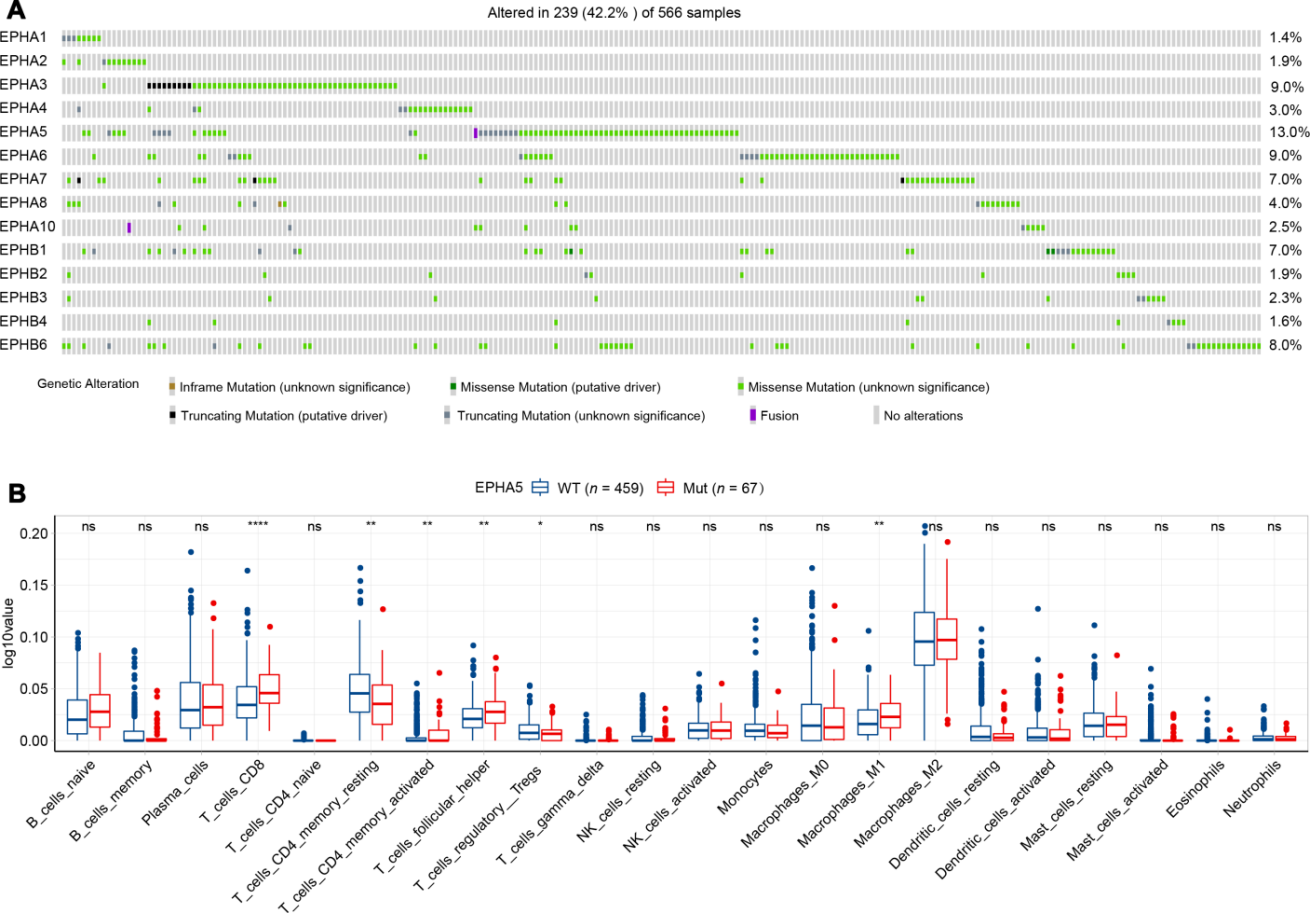
**EPHA5 mutations and immune infiltration are correlated in the TCGA LUAD cohort.** (**A**) Mutational oncoplot of the Eph family in LUAD. (**B**) Comparison of immune infiltration between EPHA5-Mut and EPHA5-WT tumors. The ordinate log10value represents log10(cibersort score+1).

Before investigating the association between Eph receptors and antitumor immunity, we first determined the mutational landscape of different Eph receptors in LUAD patients in the TCGA database. As shown in Figure 1, 239/566 patients had mutations in Eph family members. EPHA5 was the most frequently mutated gene (13%), followed by EPHA3 (9%). The mutation frequencies of other Eph family members were lower than 10% (Figure 1A). To ensure that a sufficient number of patients with mutations were included in subsequent statistical analyses, we selected EPHA5, with a mutation frequency of higher than 10%, to examine the relationship of EPHA5 mutations with tumor immune microenvironment.

**Figure 2:**
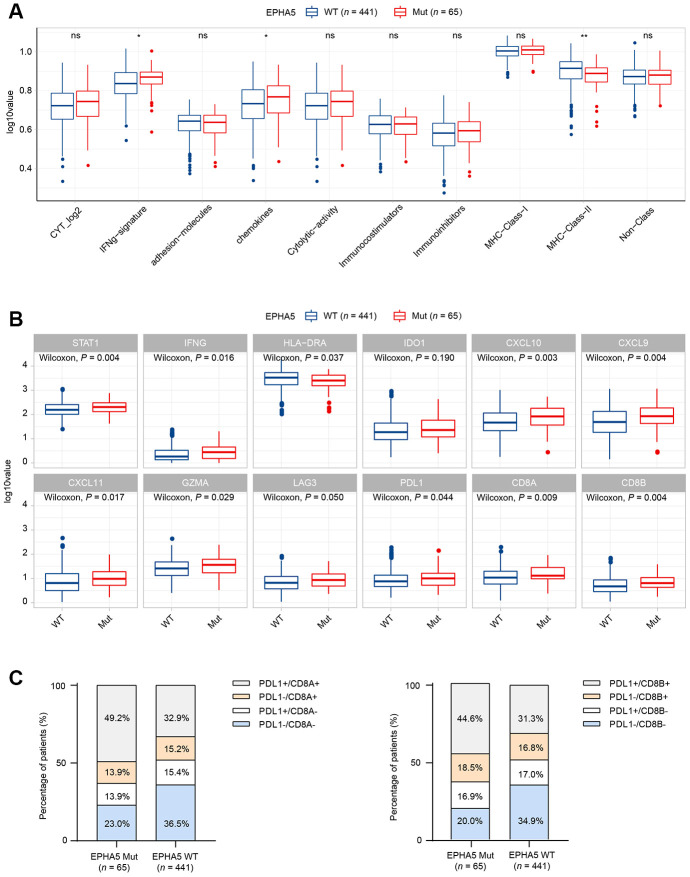
**Alterations in EPHA5 are associated with enhanced immunity in the TCGA LUAD cohort.** (**A**) IFN-γ and chemokine signatures were enriched in EPHA5-Mut tumors. The ordinate log10value represents log10(ssGSEA score+1). (**B**) The expression levels of immune checkpoint inhibitor genes were significantly higher in EPHA5-Mut tumors than in EPHA5-WT tumors. The ordinate log10value represents log10(TPM+1). (**C**) A higher proportion of PDL1^+^/CD8^+^ cells was observed in EPHA5-Mut tumors than in EPHA5-WT tumors.

TILs play an essential antitumor role in the TME. CIBERSORT analysis of the RNA-seq data from the TCGA LUAD dataset showed increased recruitment of CD8^+^ T cells and M1 macrophages and reduced recruitment of immunosuppressive cells, e.g., regulatory T cells (Tregs), in EPHA5-Mut samples (Figure 1B). Next, we evaluated the association between EPHA5 mutations and immune signatures. As shown in Figure 2A, EPHA5 mutations were correlated with significantly increased expression of the interferon-gamma (IFN-γ) signature genes as well as the chemokine signature genes, which has been suggested to be related to the immunotherapy response [18]. Among the 6 genes in the IFN-γ signature, the expression levels of only IDO1 and HLA-DRA were not distinct; those of the other 4 genes—IFN-γ and the chemokines CXCL9, CXCL10, and CXCL11were higher in EPHA5-Mut samples. Additionally, the expression of GZMA, an important factor in the cytolytic activity signature, had more abundance in EPHA5-Mut samples. Notably, we evaluated the expression of immune checkpoint genes and found that the expression of inhibitory checkpoint molecules, such as LAG3 and PDL1 (CD274), was higher in the EPHA5-Mut group than in the EPHA5-WT group (Figure 2B).

The TME has been proposed to be classified into four types based on the presence of PDL1 (CD274) and CD8^+^ TILs [19]. Samples in which the PDL1 and CD8A/CD8B expression levels above the corresponding median RNA expression levels were defined as positive. The EPHA5-Mut group contained a higher proportion of PDL1^+^/CD8^+^ samples than the EPHA5-WT group (Figure 2C), indicating that the EPHA5-Mut population is more likely to benefit from immune checkpoint blockade (ICB) therapy than the EPHA5-WT population.

**Figure 3:**
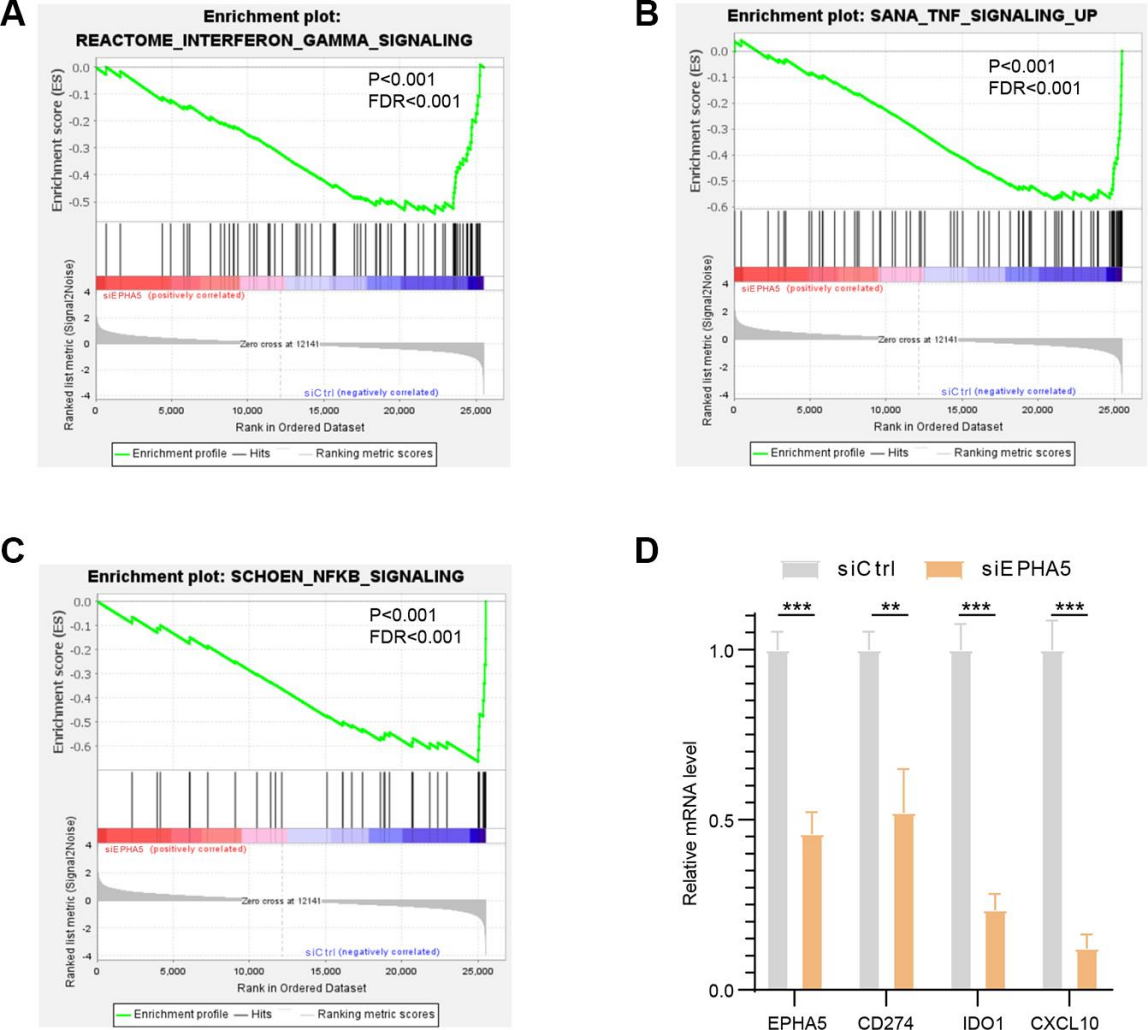
**Knockdown of EPHA5 in H1299 cells impaired immune related pathways enrichment and expression of immune checkpoint genes.** EPHA5 depletion in H1299 cells reduced the enrichment of (**A**) IFN-γ, (**B**) TNF, and (**C**) NF-κB signaling pathway components. (**D**) EPHA5 KD in H1299 cells downregulated the expression of PDL1 (CD274), IDO1 and CXCL10. **: *P* < 0.01, ***: *P* < 0.001.

To further elucidate the association between EPHA5 and antitumor immunity, we knocked down the expression of EPHA5 in H1299 cells. siRNA-mediated depletion of EPHA5 in H1299 cells decreased the enrichment of interferon-γ (IFN-γ) and the nuclear factor kappa B (NF-κB) signaling pathway components which are responsible for the expression of PDL1 (CD274) [20, 21] (Figure 3A–3C). Further real-time PCR analysis revealed that knockdown of EPHA5 in H1299 cells downregulated the expression of PDL1 (CD274), IDO1 and CXCL10 (Figure 3D).

### EPHA5 mutations are associated with elevated TMB and TNB in LUAD.

**Figure 4:**
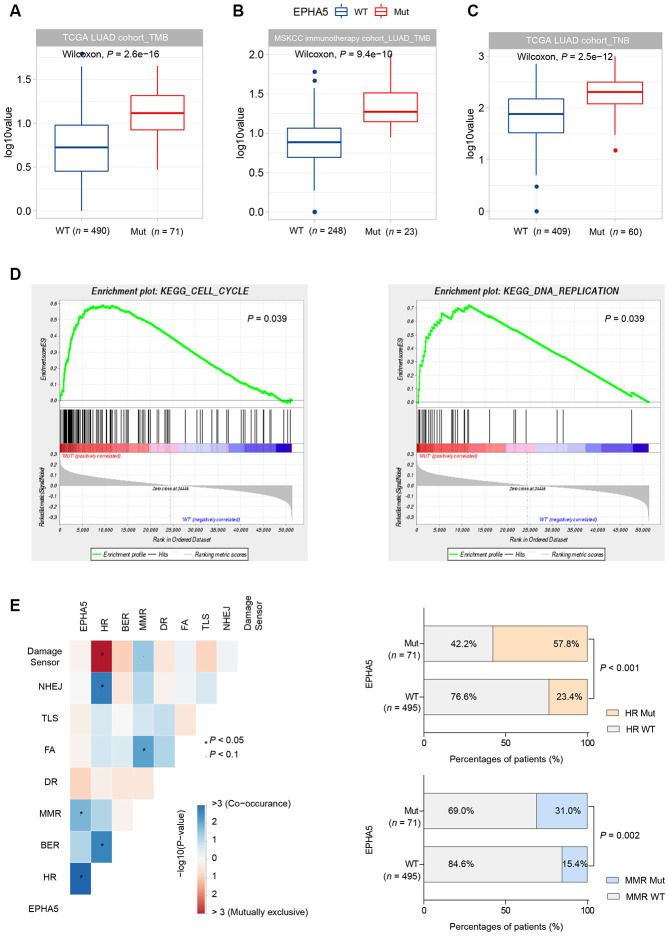
**EPHA5 mutations are related to increased TMB and TNB in LUAD.** EPHA5-Mut tumors had a markedly higher TMB than EPHA5-WT tumors in both the (**A**) TCGA LUAD cohort and (**B**) MSKCC immunotherapy LUAD cohort. The ordinate log10value represents log10(TMB+1). (**C**) In the TCGA LUAD cohort, EPHA5-Mut tumors had an increased immunogenic TNB. The ordinate log10value representslog10(TNB+1). (**D**) EPHA5 mutations were positively correlated with enrichment of the cell cycle and DNA replication pathways in the TCGA LUAD cohort. (**E**) Significantly increased mutation frequencies of genes in the HR and MMR pathways were observed in EPHA5-Mut tumors in the TCGA LUAD cohort.

TMB has been reported to be associated with various immune signatures [22]. Given the evidence that EPHA5 mutations were associated with the tumor immune microenvironment, we sought to determine whether EPHA5 mutations are correlated with TMB. In the TCGA LUAD cohort, EPHA5-Mut patients had a significantly higher TMB than EPHA5-WT patients (Figure 4A). Similarly, in the Memorial Sloan Kettering Cancer Center (MSKCC) immunotherapy cohort (Supplementary Table 1), EPHA5-Mut LUAD cancers also exhibited a significantly higher TMB than EPHA5-WT LUAD cancers, indicating a convincing association between EPHA5 mutations and TMB (Figure 4B).

Neoantigens can be derived from tumor somatic mutations. Considering that EPHA5-Mut patients had a higher number of somatic mutations, we assessed TNB in different groups. Consistent with the pattern of somatic mutations, an immunogenic TNB was more common in patients with EPHA5 mutations than in patients without EPHA5 mutations (Figure 4C). This difference may explain the more abundant infiltration of TILs in the EPHA5-Mut TME than in the EPHA5-WT TME, as these mutated neoantigens can be recognized by TILs and induce tumor-specific immune responses.

Uncontrolled cell cycle progression induced by oncogene hyperactivation and DNA replication stress causes replication fork arrest and DNA damage [23]. Combined with DDR deficiency, unrepaired DNA damage tends to generate tumor mutations. Herein, gene set enrichment analysis (GSEA) revealed that EPHA5 mutations were accompanied with accelerated cell cycle progression and increased DNA replication (Figure 4D). Additionally, we discovered significantly increased mutation frequencies for genes in the homologous recombination repair (HR) and mismatch repair (MMR) pathways (Figure 4E, Supplementary Table 2).

**Figure 5:**
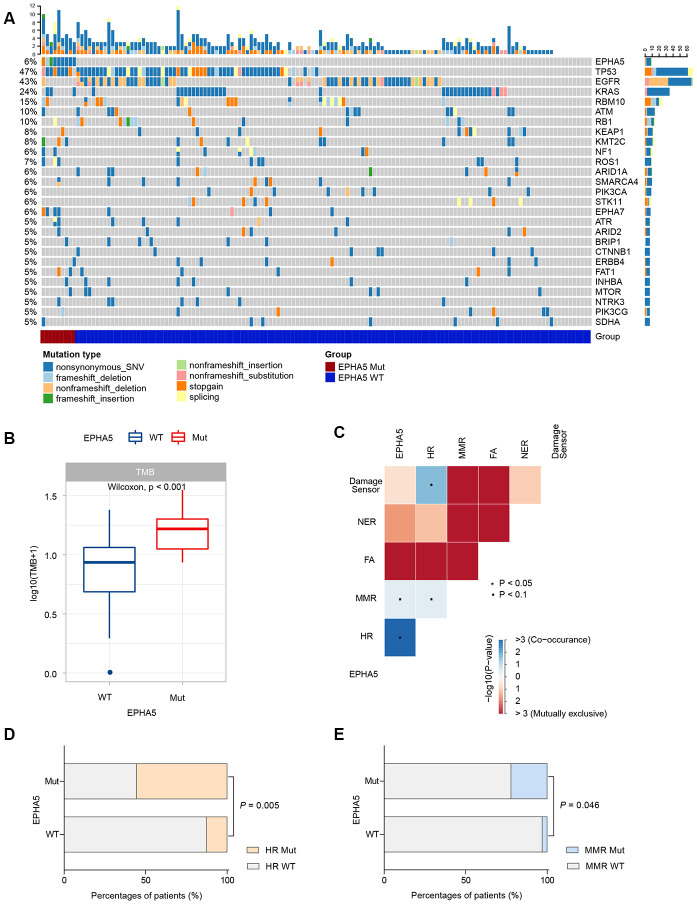
**EPHA5 mutation is correlated with increased TMB and occurred concurrently with DNA repair pathways in Chinses LUAD patients.** (**A**) Mutational landscape of the Chinese LUAD cohort. (**B**) EPHA5-Mut tumors had a markedly higher TMB than EPHA5-WT tumors. (**C**) EPHA5 mutations occurred concurrently with HR and MMR pathways. Markedly higher proportion of (**D**) HR and (**E**) MMR pathway mutations were observed in EPHA5-Mut group.

Then, we collected the formalin-fixed, paraffin-embedded (FFPE) samples of 143 Chinese LUAD patients and performed targeted DNA sequencing with a 543-gene panel. Analysis of the mutational profile showed that the frequency of EPHA5 mutations (6.3%, 9/143, Figure 5A) in the Chinese cohort was slightly lower than that in the TCGA cohort and the MSKCC immunotherapy cohort. Despite the low EPHA5 mutation frequency in the Chinese cohort, patients with EPHA5 mutations had a significantly higher TMB (range from 0 to 34.3 mutations/Mb) than patients with WT EPHA5 (Figure 5B). Similarly, mutations in HR or MMR pathway genes occurred concurrently with EPHA5 (Figure 5C–5E), indicating that EPHA5 mutations accompanied by deficiencies in DNA repair pathways tend to cause accumulation of mutations in tumors.

### EPHA5 mutations are associated with favorable prognosis after ICB therapy in patients with LUAD

**Figure 6:**
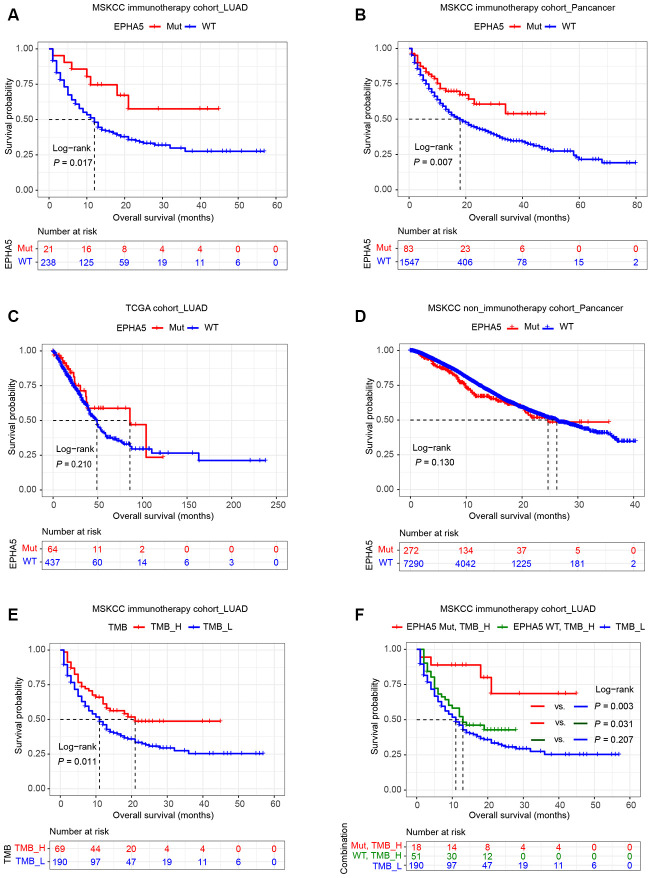
**Relation of EPHA5 to clinical outcomes in LUAD.** Patients with tumors harboring EPHA5 mutations had longer OS times than those with tumors without EPHA5 mutations in the MSKCC immunotherapy cohort: (**A**) LUAD set, (**B**) pancancer set. The OS time did not differ significantly between EPHA5-Mut and EPHA5-WT patients not treated with immunotherapy in the (**C**) TCGA LUAD or (**D**) MSKCC pancancer cohort. (**E**) High tumor mutation burden (TMB_H) was positively correlated with prolonged OS in the MSKCC immunotherapy cohort. (**F**) Survival curves were generated for patients in the MSKCC immunotherapy cohort stratified by both the EPHA5 mutation status and the TMB. *P*-values calculated with the log-rank test are shown.

High PDL1 expression, high TMB, high CD8^+^ T cell infiltration and the IFN-γ signature are well-documented predictive biomarkers for the response to immunotherapy [8]. The associations between EPHA5 and these biomarkers indicated that EPHA5 mutation alone is likely a novel predictor for the response to ICB therapy. Thus, we examined this hypothesis in the MSKCC immunotherapy cohort of LUAD patients. Consistent with the results described above, EPHA5-Mut patients had significantly more favorable overall survival (OS) than EPHA5-WT patients after immunotherapy (Figure 6A). Similarly, in the MSKCC pancancer cohort of patients treated with immunotherapy, EPHA5-Mut patients had significantly more favorable OS than EPHA5-WT patients (Figure 6B). However, in the cohorts of patients from the TCGA LUAD and MSKCC pancancer cohorts who were not treated with immunotherapy, no significant difference in OS was observed between EPHA5-Mut and WT patients (Figure 6C, 6D), suggesting that the prognostic value of EPHA5 mutation is specific for patients treated with immunotherapy. In another advanced non-squamous NSCLC cohort of patients treated with immunotherapy (Hellmann Cohort [24], Supplementary Table 3), all patients with EPHA5 mutation (nonsynonymous_snv) presented durable clinical benefit (Supplementary Figure 1A) and had high TMB (TMB > 314 mutations, i.e., the top 25% of patients, Supplementary Figure 1B). Moreover, the EPHA5-Mut group had and tended to have longer progression-free survival times (Supplementary Figure 1), further confirming the role of EPHA5 mutation as a potential prognostic factor for the response to immunotherapy.

Additionally, in the MSKCC immunotherapy cohort, patients with high TMB (TMB > 10.82 mutations/Mb, i.e., the top 25% of patients) had more favorable OS than those with low TMB (Figure 6E). However, higher TMB did not confer longer OS times than low TMB in patients without EPHA5 mutation (Figure 6F). Conversely, patients with both high TMB and mutation of EPHA5 had the best outcomes in terms of the response to immunotherapy, suggesting that the combination of EPHA5 mutation and high TMB is a more precise biomarker than either alone for selecting the patients most likely to benefit from immunotherapy.

## DISCUSSION

The Eph family of receptors is the largest family of RTKs. Overexpression or hyperactivation of Eph receptors has been found in cells from various cancers [25–27]. Most previous studies have focused on the role of Eph in regulating intrinsic characteristics that facilitate cancer progression. Less is known about the association of Eph receptors on antitumor immunity. Our study, for the first time, addressed the association between Eph receptor mutations and the tumor immune microenvironment via integrated analysis of WES and RNA-seq data from the TCGA LUAD cohort. Our analysis showed that EPHA5 mutation has a significant positive association with antitumor immunity, including increased infiltration of adaptive and innate lymphocytes and enriched IFN-γ and cytolytic activity signatures. Moreover, EPHA5-Mut LUAD tumors displayed higher TMB than EPHA5-WT tumors in both the TCGA and Chinese cohorts. Consistent with these preclinical predictions, clinical analysis based on the MSKCC immunotherapy cohort confirmed the potential of EPHA5 mutation as a prognostic biomarker for the immunotherapy response.

The “cancer-immunity cycle” proposed by Chen and Mellman begins with the release of tumor cell antigens [28]. Among the Eph receptor family members, EphA2 and EphA3 have been identified as tumor-associated antigens (TAAs), and their epitopes can be recognized by both CD4^+^ and CD8^+^ T cells [29, 30]. EphA2 has also been reported to regulate immune cell trafficking. Two other receptors, EphA1 and EphA4, are expressed in T cells and mediate T cell chemotaxis *in vitro* [31–33]. Although no evidence currently suggests that EPHA5 is a TAA, our results showed that mutations in EPHA5 were associated with increased TMB and TNB. During the subsequent steps of the cancer-immunity cycle, EPHA5 mutations likely do not correlate with TAA presentation, as the MHC-I signature did not differ between patients with WT EPHA5 and those with mutated EPHA5. However, components of the chemokine signature, including CXCL9, CXCL10 and CXCL11, which are responsible for immune cell migration, differentiation, and activation [34], were enriched in EPHA5-Mut patients, leading to infiltration of cytotoxic T cells. More importantly, EPHA5 mutations were correlated with high expression levels of checkpoint inhibitors such as LAG3 and PDL1, suggesting that mutations in EPHA5 were accompanied by CD8^+^ T cell exhaustion in LUAD. Notably, we validated the impact of EPHA5 on the expression of PDL1, IDO and CXCL10 in H1299 cells. Thus, for the first time, we identified the link between EPHA5 and the tumor immune microenvironment in LUAD. However, this relationship requires further validation in mouse models.

Numerous studies have demonstrated that immunotherapy, especially with inhibitors of PD-1 or PDL1, has improved the prognosis of NSCLC patients and reformed therapeutic strategies for NSCLC [4, 7, 35]. Currently, several biomarkers that predict drug sensitivity or resistance have been identified, including TMB, CD8^+^ T cell infiltration into the TME and intratumoral PDL1 expression as evaluated by immunohistochemistry (IHC) [8]. Although the detection of biomarkers can identify patients who may benefit from immunotherapy, not all patients with high TMB or high expression of PDL1 respond well [36]. Other challenges, such as appropriate definition of cutoff values, limit the clinical application of these biomarkers. Thus, identification of biomarkers representing two or more of the above factors is needed to guide immunotherapy applications. Our findings that EPHA5 mutation was correlated with high TMB, high PDL1 expression, high CD8+ cell infiltration and high expression of IFN-γ signature components in LUAD imply that EPHA5 mutations can predict most of the current features related to the immunotherapy response in LUAD.

Moreover, we found a higher proportion of TIL^+^PD-L1^+^ patients among EPHA5-Mut patients than among EPHA5-WT patients, supporting the conclusion that EPHA5-Mut patients treated with PDL1 blockade therapy are likely to exhibit a relatively good prognosis. Most importantly, although patients with high TMB (25% top) had longer OS times than patients with low TMB, the subgroup of EPHA5-WT patients with high TMB had OS times equivalent to those of patients with low TMB. Thus, detection of the EPHA5 mutation status may help prevent overtreatment of EPHA5-WT patients with high TMB and avert the development of immune-related adverse effects in these patients.

This study has several limitations. First, we established the relationship between EPHA5 and antitumor immunity based only on data from tumor specimens. More in-depth studies using mouse model- or cell culture-based techniques are needed to confirm our findings. Second, validation of the prognostic or predictive role of EPHA5 mutations for the immunotherapy response is necessary in an independent cohort of ICB-treated patients. In conclusion, we discovered the obvious importance of EPHA5 mutations in increasing TMB, T cell infiltration into the TME and PDL1 expression, implying that EPHA5 mutation is a potential prognostic marker for the immunotherapy response.

## MATERIALS AND METHODS

### Data acquisition

Three LUAD genomic datasets were utilized in this study: the genomic dataset from the MSK-IMPACT clinical sequencing cohort pancancer study (MSKCC, Nat Med 2017), a LUAD dataset (TCGA, PanCancer Atlas) and an MSKCC immunotherapy cohort (Nat Genet 2019). The gene expression profiling data for the LUAD dataset (TCGA, PanCancer Atlas) were downloaded from the GDC data portal (https://portal.gdc.cancer.gov/). Fragments per kilobase million mapped reads (FPKM) values in RNA-seq data were transformed into transcripts per kilobase million (TPM) values.

### Evaluation of immune cell infiltration and immune gene signatures

The abundances of 22 immune cell types were calculated with CIBERSORT [37]. The gene sets for cytolytic activity (granzyme-A and perforin-1), the IFN-γ signature, immune costimulators, immune inhibitors, chemokines, the HLA-I signature (MHC-class I), and the HLA-II (MHC-class II) signature were analyzed as described in previous studies [18, 38].

TNB and TMB data were obtained from published TCGA data [39]. Somatic alterations in ten oncogenic signaling pathways were determined as described in previously published literature [40].

### Patient information

A total of 143 LUAD patients were recruited from The First Affiliated Hospital of Soochow University. Clinically recorded information, such as age, sex, smoking status, and pathologic stage, was collected as shown in Supplementary Table 4. All patients provided written informed consent for molecular analysis of their tissue samples.

This study was approved by the Ethics Committee of the First Affiliated Hospital of Soochow University. All procedures in studies involving human participants were performed in accordance with the ethical standards of the institutional and/or national research committee and with the 1964 Helsinki Declaration and its later amendments or with comparable ethical standards.

### DNA extraction and sequencing

FFPE tumor specimens and matched blood samples were collected and submitted to construct the designed 543-gene NGS panel (Supplementary Table 5) for testing based on next-generation sequencing (NGS). DNA extraction and sequencing were performed using standard protocols described previously [41].

### Variant analysis

To identify somatic single nucleotide variants (SNVs) and indels, matched blood samples from patients were used as controls for the FFPE tumor samples with mutations. SNVs were called with VarDict (version 1.5.1) [42]. Variants were annotated with ANNOVAR [43]. Variants that met one or more of the following criteria were excluded: (a) < 30× sequencing coverage, (b) silent mutations in nonreference alleles, (c) < 5 supporting reads, (d) an allele frequency of ≥0.005 in the Exome Aggregation Consortium (ExAC) or Genome Aggregation Database (gnomAD), or (e) an allele frequency of <0.02 in the tumor sample. TMB (mutations/Mb) was calculated with the previously reported algorithm [44].

### Cell culture and transfection

H1299 cells were grown in RPMI 1640 medium (Gibco) supplemented with 10% FBS and 1% penicillin-streptomycin (Invitrogen). Cells were maintained at 37° C in an atmosphere containing 5% CO_2_.

Transient knockdown of the Epha5 gene was performed by transfection of siRNA with Lipofectamine® RNAiMAX Transfection Reagent (#13778150, Invitrogen). The siRNA (siEPHA5) targeted the following sequence: catctccagtcaccaatgtga.

### RNA isolation and RT-qPCR

Cultured cells were lysed with TRIzol^TM^ Reagent (#15596018, Invitrogen). Total RNA was isolated according to the manufacturer’s instructions. mRNA was reverse transcribed to cDNA with a cDNA synthesis kit (AE311-03, TransGen Biotech, Beijing, China). RT-qPCR was performed with diluted cDNA (1:4) in three wells per reaction with the appropriate primer pair and SYBR green master mix (Bio-Rad) in a Bio-Rad iCycler iQ Real Time PCR System. All RT-qPCR experiments were repeated at least three independent times. The primer sequences are listed below: Human IDO1-F: GCCAGCTTCGAGAAAGAGTTG; Human IDO1-R: ATCCCAGAACTAGACGTGCAA; Human CD274-F: TGGCATTTGCTGAACGCATTT; Human CD274-R: TGCAGCCAGGTCTAATTGTTTT; Human CXCL10-F: GTGGCATTCAAGGAGTACCTC; Human CXCL10-R: TGATGGCCTTCGATTCTGGATT; Human EPHA5-F: GTGACCGATGAACCTCCCAAA; Human EPHA5-R: CCAGGTCTGCACACTTGACAG; Human beta-Actin-F: TGACAGGATCGAGAAGGAGA; Human beta-Actin-R: CGCTCAGGAGGAGCAATG.

### RNA sequencing

RNA was quantified using a Life Invitrogen Qubit 3.0/4.0 fluorometer and assessed using an Agilent 2100 Bioanalyzer system. Then, 8 μl of total RNA was used as input with a SMARTer Stranded Total RNA-Seq Kit v2 (Takara) in accordance with the low-throughput protocol. After PCR enrichment and purification of adapter-ligated fragments, the library with adapters was analyzed with the Life Invitrogen Qubit 3.0/4.0 and assessed in the Agilent 2100 Bioanalyzer system. Then, RNA-seq was performed on a Illumina NovaSeq 6000 Sequencing System. To ensure data quality, raw reads were preprocessed by removing low-quality sequences, removing junction contamination (with Trimmomatic) [45], detecting the A/T/G/C content distribution (with RSeQC) [46], removing rRNA (with bowtie2) [47], etc., to obtain high-quality sequences (clean reads). All subsequent analyses were based on clean reads. Reference gene and genome annotation files were downloaded from the GENCODE website (https://www.gencodegenes.org/human/). Clean data were aligned to the reference genome with HISAT [48] (http://ccb.jhu.edu/software/hisat2/index.shtml). FeatureCounts was used to estimate the expression level of each gene. Gene expression was quantified with the FPKM method [49].

### GSEA

GSEA (http://software.broadinstitute.org/gsea/index.jsp) was conducted based on the expression results by using the default parameters on c2 gene sets in the Molecular Signatures Database (MSigDB) (http://software.broadinstitute.org/gsea/msigdb).

### Statistical analysis

For two-group comparisons, statistical significance was estimated with unpaired Student’s t tests for normally distributed variables and with the Mann-Whitney U test for nonnormally distributed variables. Fisher’s exact test for pairwise comparisons was performed to detect sets of pathways mutually exclusive or cooccurring with EPHA5. Fisher’s exact test was also used to compare the proportions of mutations in HR and MMR genes between the EPHA5-Mut and EPHA5-WT groups. The Kaplan-Meier method was used to generate survival curves for the subgroups in each dataset, and the log-rank (Mantel-Cox) test was used to determine the statistical significance of the differences. Statistical analyses were performed in R (https://www.r-project.org/, version 3.6.1), and *P* < 0.05 was considered to indicate a statistically significant difference.

### Ethics approval

This study was performed in accordance with the ethical standards and the Declaration of Helsinki and according to national and international guidelines. Surgically procured tumor samples from patients were obtained in the Department of Thoracic Surgery, The First Affiliated Hospital of Soochow University with informed patients’ consent for research purposes.

## Supplementary Material

Supplementary Figure 1

Supplementary Tables
