# Supplementary material for: EPHA5 mutations predict survival after immunotherapy in lung adenocarcinoma
Source: Aging (Albany NY). 2020 Dec 3;13(1):598–618. doi: 10.18632/aging.202169 (PMC7834994; doi:10.18632/aging.202169)
Supplement: Supplementary Figure 1 [file aging-13-202169-s001.pdf]

SUPPLEMENTARY FIGURE

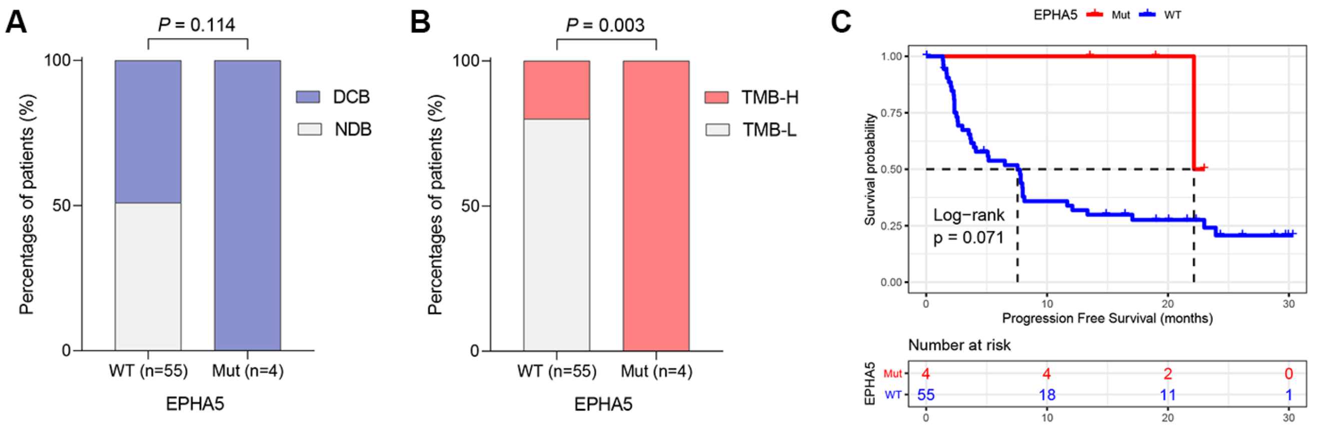

Supplementary Figure 1. Non-squamous NSCLC patients with EPHA5 mutation had better clinical response and outcome after ICI therapy. All patients with EPHA5 mutations (**A**) presented durable clinical benefit to ICIs, and (**B**) had high TMB. (**C**) The EPHA5-Mut group tended to have longer PFS than the EPHA5-WT group.
