# Supplementary material for: EPHA5 mutations predict survival after immunotherapy in lung adenocarcinoma
Source: Aging (Albany NY). 2020 Dec 3;13(1):598–618. doi: 10.18632/aging.202169 (PMC7834994; doi:10.18632/aging.202169)
Supplement: Supplementary Tables [file aging-13-202169-s002.pdf]

SUPPLEMENTARY TABLES

Supplementary Table 1. Clinical characteristics of the studied LUAD patients from Hellmann cohort.

Characteristics	All patients	EPHA5 Mut	EPHA5 WT	P value
Total (cases)	59	4	55	
**Gender** [cases (%)]				0.624
Male	22 (37.3)	2 (50.0)	20 (36.4)	
Female	37 (62.7)	2 (50.0)	35 (63.6)	
**Age** (years)				0.148
Median	63	56.5	66	
IQR	56.00-70.50	52.25-61.25	56.50-71.00	
**Smoking status** [cases (%)]				0.566
Current/former	46 (78.0)	4 (100.0)	42 (76.4)	
Never	13 (22.0)	0 (0.0)	13 (23.6)	
**Performance status** [cases (%)]				1
ECOG_0	28 (47.5)	2 (50.0)	26 (47.3)	
ECOG_1	31 (52.5)	2 (50.0)	29 (52.7)	
**Clinical benefit** [cases (%)]				0.114
DCB	31 (52.5)	4 (100.0)	27 (49.1)	
NDB	28 (47.5)	0 (0.0)	28 (50.9)	

DCB: durable clinical benefit, NDB: no durable benefit;
P value: Wilcoxon test rank sum or Fisher's exact test (two sided) was used for the comparison between the EPHA5 Mut and WT groups.

Supplementary Table 2. DDR core pathway membership.

Base Excision Repair (BER)	Nucleotide Excision Repair (NER, including TC-NER and GC-NER))	Mismatch Repair (MMR)	Fanconi Anemia (FA)	Homologous Recombination (HR)	Non-homologous End Joining (NHEJ)	Direct Repair (DR)	Translesion Synthesis (TLS)	Damage Sensor etc.
PARP1	CUL5	EXO1	FANCA	MRE11A	LIG4	ALKBH2	POLN	ATM
POLB	ERCC1	MLH1	FANCB	NBN	NHEJ1	ALKBH3	POLQ	ATR
APEX1	ERCC2	MLH3	FANCC	RAD50	POLL	MGMT	REV1	ATRIP
APEX2	ERCC4	MSH2	FANCD2	TP53BP1	POLM		REV3L	CHEK1
FEN1	ERCC5	MSH3	FANCI	XRCC2	PRKDC		SHPRH	CHEK2
TDG	ERCC6	MSH6	FANCL	XRCC3	XRCC4			MDC1
TDP1	POLE	PMS1	FANCM	BARD1	XRCC5			RNMT
UNG	POLE3	PMS2	UBE2T	BLM	XRCC6			TOPBP1
	XPA			BRCA1				TREX1
	XPC			BRCA2				
				BRIP1				
				EME1				
				GEN1				
				MUS81				
				PALB2				
				RAD51				
				RAD52				
				RBBP8				
				SHFM1				
				SLX1A				
				TOP3A				

Supplementary Table 3. Clinical characteristics of the studied LUAD patients from MSKCC immunotherapy cohort.

Characteristics	All patients	EPHA5 Mut	EPHA5 WT	P value
**Total** (cases)	271	23	248	
**Gender** [cases (%)]				0.827
Male	117 (43.2)	9 (39.1)	108 (43.5)	
Female	154 (56.8)	14 (60.9)	140 (56.5)	
**Age Group at Diagnosis in Years** [cases (%)]				0.55
31-50	28 (10.3)	2 (8.7)	26 (10.5)	
50-60	61 (22.5)	8 (34.8)	53 (21.4)	
61-70	85 (31.4)	6 (26.1)	79 (31.9)	
>71	97 (35.8)	7 (30.4)	90 (36.3)	
**Drug Type** [cases (%)]				0.634
PD-1/PDL-1	255 (94.1)	21 (91.3)	234 (94.4)	
Combo	16 (5.9)	2 (8.7)	14 (5.6)	

P value: Fisher's exact test (two sided) was used for the comparison between the EPHA5 Mut and WT groups

Supplementary Table 4. Clinical characteristics of the studied LUAD patients from Chinese cohort.

Characteristics	All patients	EPHA5 Mut	EPHA5 WT	P value
**Total** (cases)	143	9	134	
**Gender** [cases (%)]				0.086
Male	86 (60.1)	8 (88.9)	78 (58.2)	
Female	57 (39.9)	1 (11.1)	56 (41.8)	
**Age** (years)				0.206
Median	62.00	66.00	62.00	
IQR	55.00-68.00	63.00-67.00	54.25-68.00	
**Smoking status** [cases (%)]				0.140
Current/former	58 (40.6)	6 (66.7)	52 (38.8)	
Never	70 (49.0)	2 (22.2)	68 (50.7)	
NA	15 (10.5)	1 (11.1)	14 (10.4)	
**Pathological stage** [cases (%)]				0.921
I	16 (11.2)	1 (11.1)	15 (11.2)	
II	27 (18.9)	2 (22.2)	25 (18.7)	
III	59 (41.2)	3 (33.3)	56 (41.8)	
IV	41 (28.7)	3 (33.4)	38 (28.3)	

NA: not available;

P value: Wilcoxon test rank sum or Fisher's exact test (two sided) was used for the comparison between the EPHA5 Mut and WT groups, and NA was not included in the statistical analysis

Supplementary Table 5. Gene list of the designed NGS panel.

Hugo Symbol	Hugo Symbol	Hugo Symbol	Hugo Symbol	Hugo Symbol	Hugo Symbol	Hugo Symbol
AMER1	IPO7	CREBBP	PDCD1LG2	HSPA4	ESR1	DHFR
ASXL1	MED19	CRKL	PDGFRB	CNOT8	ETV6	GSTA1
ATRX	MARK2	CRLF2	PIK3C2G	GABRP	FANCA	SOD2
AURKB	ANO1	CSF1R	PIK3CB	F13A1	FBXW7	SLC22A2
BCOR	KDM5A	CSF3R	PIK3CG	FLOT1	FGF19	SEMA3C
BRD4	ZDHHC17	CXCR4	PIK3R1	HSPA1B	FGF3	ABCB1
CBFB	VSIG10	CYLD	PIK3R2	MAP3K4	FGF4	SLC31A1
CDC73	CTAGE5	DAXX	PLCG2	TRA2A	FGFR1	HSPA5
CDK12	PRPF39	DICER1	PPARG	ABCA13	FGFR2	CYP2C19
CDK8	MAP4K5	DIS3	PPP2R1A	CALD1	FGFR3	CYP2C8
CIC	ARID4A	DOT1L	PRKCI	UBE3C	FH	SLIT1
CTCF	TECPR2	EP300	PTCH1	MMP16	FLT1	ABCC2
FAM46C	APOPT1	EPHA3	PTPN11	RIPK2	HRAS	RRM1
FAT1	HAUS2	EPHA5	RAD51	RAD21	IDH1	GSTP1
FOXL2	ATP9B	EPHA7	RAD51B	IARS	IDH2	SLCO1B3
FUBP1	SPC24	EPHB1	RAD52	MAPKAP1	JAK1	SLCO1B1
GATA1	MYADM	ERBB4	RAD54L	UPF2	JAK2	CYP19A1
GATA2	KIR3DX1	ERG	RAF1	PDE6C	KRAS	CYBA
IKZF1	ZNF805	ETV1	RARA	ADRB1	MAP2K1	TYMS
KDM5C	C20orf96	EZH2	RPTOR	ACADSB	MDM2	CYP2B6
LMO1	NCOA6	FANCC	RUNX1	TPH1	MDM4	XRCC1
MYCL	TMPRSS15	FANCG	SDHA	PTPRJ	MEN1	ERCC2
NKX2-1	DSCAM	FAS	SETD2	FOLH1	MET	ERCC1
PRDM1	RRP1B	FGFR4	SF3B1	ALG9	MLH1	HNF4A
PREX2	C22orf23	FLCN	SMAD2	HYOU1	MRE11A	CBR3
RBM10	MOV10L1	FLT3	SMAD3	MAGOHB	MSH3	CYP2D6
RNF43	WWC3	FLT4	SMARCA4	TXNRD1	MSH6	GSTM1
SPEN	ARHGAP6	FOXP1	SMARCB1	HCAR2	MTOR	EWSR1
TET2	BCYRN1	GATA3	SMO	EXOSC8	MUTYH	FUS
TOP1	ZNF711	GLI1	SOCS1	TNFSF13B	NBN	HEY1
PDPN	TBC1D8B	GNA11	SOX2	SNX6	NF2	NR4A3
GMEB1	DOCK11	GNA13	SOX9	AK7	NOTCH1	PDGFB
MTF1	LONRF3	GNAQ	SPOP	CYFIP1	NRAS	SS18
ZZZ3	KIAA1210	GNAS	SRC	GANC	NT5C2	EML4
RABGAP1L	ARHGAP4	GRIN2A	STAT3	NEO1	NTRK1	CD74
IRF6	BAP1	GSK3B	SUFU	FANCI	NTRK2	SLC34A2
PLEKHH2	DDR2	H3F3A	SYK	ITGAL	NTRK3	KIF5B
PNO1	ERRFI1	HGF	TBX3	ARMC5	PALB2	RIC1
TSN	NPM1	HNF1A	TGFBR2	NUP93	PBRM1	AGPAT9
ARL6IP6	ROS1	ID3	TMPRSS2	IRF8	PDGFRA	CAST
NAB1	NR1I3	IGF1R	TNFAIP3	NF1	PIK3CA	ZMYM4
ALS2CR11	OTOS	IGF2	TNFRSF14	RHOT1	PMS2	SLC30A5
ULK4	C8orf34	IKBKE	TSHR	TAF15	POLD1	CEP120
KPNA4	NAB2	IL7R	U2AF1	KPNB1	POLE	C5orf15
ZBBX	PPHLN1	INHBA	WHSC1	ABCA8	PRKAR1A	SIMC1
LRRC34	ABL1	INPP4B	WHSC1L1	KCNJ2	PTEN	PGBD1
SEL1L3	AKT2	IRF4	XPO1	NUP85	RAC1	HAUS6
NFXL1	AKT3	IRS2	CXCL8	LAMA3	RAD50	ZNF367
SHROOM3	ALOX12B	JAK3	EIF4G3	MALT1	RAD51C	SFXN4
FRAS1	ARID1A	JUN	STMN1	CDC25B	RAD51D	CEP290
CBR4	ARID1B	KDM6A	LEPR	CD40	RB1	STYX
GPM6A	ARID2	KDR	ASH1L	FGF16	RET	MAPKBP1
FAM149A	ATR	KEAP1	FMO1	RPA4	RICTOR	ZNF91
MTRR	AURKA	KMT2A	SIPA1L2	STAG2	SDHB	APOL2

C5orf42	AXIN1	KMT2C	RYR2	BRS3	SDHC	NKAP
ADAMTS6	AXL	KMT2D	ADSS	IRAK1	SDHD	DBT
BTF3	BARD1	LYN	ID2	AKT1	SMAD4	DIAPH1
ANKRA2	BCL2	MAP2K2	CALM2	ALK	STK11	HLA-DRB1
CHD1	BCL2L1	MAP2K4	MSH2	APC	TERT	COL15A1
CDKL3	BTG1	MAP3K1	PAPOLG	AR	TP53	WDR5
PURA	BTK	MCL1	REL	ARAF	TSC1	MMP3
RBM27	CALR	MED12	MEIS1	ATM	TSC2	NLRP7
FAM153B	CARD11	MEF2B	MRPL19	BLM	VEGFA	DEPDC5
HNRNPH1	CASP8	MITF	SUCLG1	BRAF	VHL	DYNC2H1
ACOT13	CBL	MPL	UBE2E3	BRCA1	WT1	STARD4
PAQR8	CCND2	MYC	NDUFS1	BRCA2	PIGF	ZNF2
SASH1	CCND3	MYCN	ARPC2	BRIP1	B2M	TOE1
CNKSR3	CCNE1	MYD88	CUL3	CCND1	MAPK1	EZR
TAGAP	CD274	NFE2L2	CAB39	CDH1	MTHFR	HLA-A
TNRC18	CD79A	NFKBIA	FANCD2	CDK4	CDA	HLA-B
SUGCT	CD79B	NOTCH2	TOP2B	CDKN2A	DPYD	HLA-C
TRIM24	CDK6	NOTCH3	CCR4	CHEK2	MTR	SLC7A8
ERI1	CDKN1A	NSD1	KIT	CTNNB1	GALNT14	RFC1
TNKS	CDKN1B	P2RY8	COX18	DNMT3A	LRP2	STRBP
TMEM67	CDKN2B	PARK2	MYO10	EGFR	ATIC	PLEKHA1
RNF19A	CDKN2C	PARP1	DROSHA	ERBB2	UGT1A1	
ANKRD46	CEBPA	PAX5	CDK7	ERBB3	XPC	
C9orf72	CHEK1	PDCD1	CDO1	ERCC4	UMPS	
